# Display of Gold in Teeth

**Published:** 1898-12

**Authors:** 


ARTICLE VIII.

### DISPLAY OF GOLD IN TEETH.

Forty years ago the universal practice in filling teeth was to conceal material and never expose gold to be gazed upon, if possible to avoid it.

The practice of that day, when such men as Harris, Hayden, Maynard, Parmelee, Dwinell, Westcott, Townsend, Clark Badger, and a host of others of corresponding merit as practitioners of dentistry, were in the lead, and competent to set examples worthy of acceptance and to be strictly followed, was to treat for preservation of teeth so as to present as natural appearance as possible; that no one could from observation discern that pluggers and gold had been used in the mouth—very unlike much of the practice of the present day, when the unreasonable craze for display of gold in teeth seems to be on the increase. In many instances healthy enamel has been sacrificed (censurably) that gold could be displayed to more conspicuous advantage, with artistic surface finish, to attract attention and please the eye, and cause comment and inquiry, "Who filled your teeth? The fillings are beautiful."

The question arises, which of the two, the former or the latter practice, is best for patients, less expensive, and most to be admired? If the former, which good judgment and good taste must sanction and commend, then as a profession, let us combine in condemnation of the latter, and encourage a return to first principles in that particular, and cherish the hope that ere the space of another decade the practice of enamel-saving and gold-concealing will be more honestly and effectively practiced than at present, and the tendency will be more honestly and effectively practiced than at present, and the tendency will be onward and upward for the preservation of teeth.

In treating for the preservation of teeth, the normal outline and appearance should be preserved, if possible, regardless of the variety and fancy of patients, who have but little idea as to what line of practice is best for them and most in accord with the true line of conservatism that should rule in practice.

We must, through a true and honest line of practice, developing good results, educate the public mind on the subject, and by all means at command encourage dentists who are side-tracking to the detriment of the profession, and doing injustice to patrons, to pause and reflect, and fall into line of true practice for the better preservation of teeth and saving of precious metal.

To be able to fill teeth successfully and beautifully with gold when requisite, is a feature in practice to be admired and commended, and every man in the profession should be ambitious and strive to attain that degree of excellence in manipulative skill, but never abuse the talent.

To be a fancy filler, for notoriety and applause, is demoralizing and hurtful to the profession and very destructive to the healthy enamel, as is daily evidenced without close inspection, a practice that merits no praise, but much censure.—B. F. A., in *Dental Weekly*.

